# Relationship between triglyceride-glucose-derived obesity indices and NAFLD in adolescents: A cross-sectional study

**DOI:** 10.1097/MD.0000000000049094

**Published:** 2026-06-05

**Authors:** Wenting Deng, Jian Mei, Aiguo Wang, Qingmin Yu, Weixin Pan, Guangli Ding, Hongli Zhou, Ashanjiang Aniwan, Bichao Yu

**Affiliations:** a Department of Geriatrics, Jinyun County People’s Hospital, Zhejiang, China; b Department of Endocrinology, Jinyun County People’s Hospital, Zhejiang, China.

**Keywords:** NAFLD, NHANES, TyG-BMI, TyG-WC, TyG-WHtR, TyG-WWI

## Abstract

Obesity and insulin resistance may lead to nonalcoholic fatty liver disease (NAFLD) in adolescents. Triglyceride-glucose-derived obesity indices reflect the combination of insulin resistance and metabolic disorders. Research on the relationship between adolescent NAFLD and triglyceride glucose–waist circumference (TyG-WC), triglyceride glucose–waist-to-height ratio (TyG-WHtR), triglyceride glucose–body mass index (TyG-BMI), and triglyceride-glucose weight-adjusted waist index (TyG-WWI) remains extremely limited. This study utilized data from the 2017 to 2020 National Health and Nutrition Examination Survey, which involved individuals aged 12 to 19 years. Two groups of participants were created based on the controlled attenuation parameter obtained from liver ultrasound transient elastography: those diagnosed with NAFLD and those without NAFLD. The study examined the relationship between adolescent NAFLD and the triglyceride-glucose (TyG) composite body measurement indicator using multivariate logistic regression, smooth curve fitting, and receiver operating characteristic. A total of 678 subjects were included, comprising 115 cases in the NAFLD group and 563 cases in the non-NAFLD group. The fully adjusted multivariate logistic models TyG-WC, TyG-WHtR, TyG-BMI, and TyG-WWI all showed significant positive correlations with NAFLD (*P* < .001). Specifically, each unit increase in TyG-WHtR was associated with a 6-fold increase in the risk of fatty liver disease (95% confidence interval: 6.03 [3.82–9.52]). The top 3 area under the curve values were obtained by TyG-WC (0.8833), TyG-WHtR (0.8761), and TyG-BMI (0.875). TyG-WC, TyG-WHtR, TyG-BMI, and TyG-WWI are significantly associated with NAFLD risk in adolescents aged 12 to 19 years and may hold clinical value for predicting NAFLD in this population.

## 1. Introduction

The rising rates of adolescent obesity and metabolic syndrome have made nonalcoholic fatty liver disease (NAFLD) the most common chronic liver disease among adolescents worldwide. The interaction between adolescent obesity, insulin resistance, and fat accumulation in the liver has significantly increased the prevalence of NAFLD in this age group.^[1–3]^ Many adolescents only discover NAFLD when they seek medical attention for severe obesity or impaired liver function. NAFLD is often asymptomatic, yet as metabolic disorders progress, it may advance to liver fibrosis, cirrhosis, end-stage liver disease, and even require liver transplantation.^[4,5]^

Insulin resistance promotes hepatic lipid synthesis and inhibits fatty acid oxidation, leading to fat accumulation in hepatocytes.^[3,6]^ This process stimulates the release of free fatty acids from adipose tissue, thereby increasing the lipid burden on the liver. Obesity leads to excessive energy conversion into fatty acids deposited in the liver.^[7,8]^ It causes abnormal production of inflammatory factors, resulting in steatohepatitis. Obesity disrupts the gut microbiota, allowing endotoxins to enter the bloodstream and trigger hepatitis.^[9,10]^ TyG assesses insulin resistance but does not account for obesity. Composite indicators such as triglyceride glucose–waist circumference (TyG-WC), triglyceride glucose–waist-to-height ratio (TyG-WHtR), triglyceride glucose–body mass index (TyG-BMI), and triglyceride-glucose weight-adjusted waist index (TyG-WWI) demonstrate significant value in evaluating insulin resistance.^[11,12]^

Some research has investigated the link between various indicators (TyG, TyG-WHtR, TyG-WC, TyG-BMI) and adult NAFLD.^[13,14]^ Research on adolescents aged 12 to 19 years remains scarce, yet NAFLD has emerged as a significant adolescent health issue. Given the substantial variability and high plasticity of obesity indicators in this population, early identification and intervention could help reduce disease incidence. This study utilized controlled attenuation parameter (CAP) obtained through liver ultrasound transient elastography to diagnose NAFLD.^[1]^ Here, we evaluated the relationship and predictive value of TyG-WC, TyG-WHtR, TyG-BMI, TyG-WWI, and adolescent NAFLD.

## 2. Methods

### 2.1. Data source and study population

**Figure 1.**
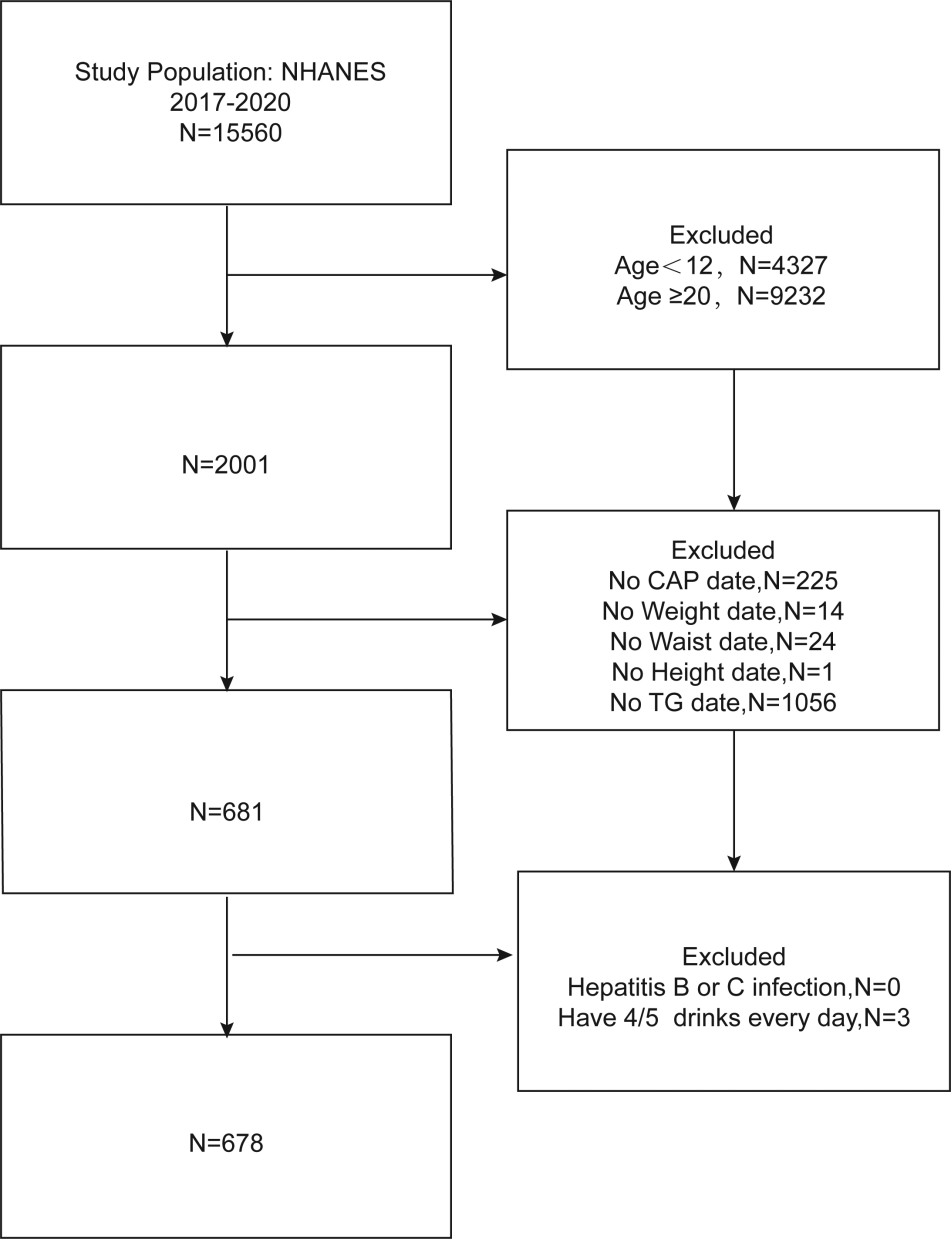
Flowchart of participant selection. CAP = controlled attenuation parameter, NHANES = National Health and Nutrition Examination Survey, TG = triglyceride.

The National Health and Nutrition Examination Survey (NHANES) database is publicly available and has obtained informed consent from all participants; therefore, this study does not require ethical review. A total of 15,560 individuals from the 2017 to 2020 NHANES database were selected. Exclusion criteria were the following: age <12 years or ≥20 years; incomplete liver ultrasound transient elastography or missing CAP and liver stiffness measurement (LSM) values due to noncompliance with liver ultrasound transient elastography criteria; missing physical measurements (e.g., weight, height, waist circumference); missing triglyceride (TG) values; positive hepatitis B surface antigen or hepatitis C antibody; consuming 4 or more alcoholic drinks daily.^[15,16]^ Following the aforementioned screening process, a total of 678 research subjects were ultimately selected (Fig. 1).

### 2.2. NAFLD assessment and TG-glucose-derived obesity indices

Liver ultrasound transient elastography detected a CAP ≥274 dB/m, indicating the presence of NAFLD. Based on NHANES database records, TyG and TyG-composite anthropometric indicators were calculated from TGs, fasting blood glucose (FBG), BMI, waist circumference (WC), height, and weight. Formulas: TyG = Ln(TG (mg/dL) × FBG (mg/dL)/2); TyG-WHtR = TyG × WHtR (WHtR = WC/Height); TyG-BMI = TyG × BMI; TyG-WC = TyG × WC; TyG-WWI = TyG × WWI (WWI = WC divided by the square root of weight).^[17–19]^

### 2.3. Covariables

Age, gender (male or female), race (Mexican American, Other Hispanic, Non-Hispanic White, Non-Hispanic Black, Other Race), income-to-poverty ratio, smoking status (defined as Yes if having smoked at least 100 cigarettes in a lifetime, otherwise No), high-density lipoprotein, uric acid, aspartate aminotransferase, alanine aminotransferase, alkaline phosphatase, LSM, C-reactive protein, poverty-to-income ratio, and low-density lipoprotein cholesterol. You can find the measuring procedure at www.cdc.gov/nchs/nhanes.

### 2.4. Statistical analysis

This study employed EmpowerStats version 2.0 (X&Y Solutions) for statistical analysis. Continuous variables are presented as mean ± standard deviation, while categorical variables are expressed as percentages. This cross-sectional study investigated the relationship between adolescent TyG-WC, TyG-WHtR, TyG-BMI, TyG-WWI, and NAFLD through multivariate logistic regression analysis, smoothing curve fitting, and receiver operating characteristic curves. *P* values <.05 were considered statistically significant, while *P* values <.001 indicated high statistical significance.

## 3. Results

### 3.1. Study population characteristics

The total cohort consisted of 678 individuals, with a mean age of 15.47 ± 2.21 years, 52.21% of whom were male. Mean TyG-WC values were 670.04 ± 155.46, mean TyG-WHtR values were 4.05 ± 0.93, mean TyG-BMI values were 200.12 ± 62.26, and TyG-WWI values were 81.14 ± 9.66. Table 1 shows the data divided into 2 groups based on NAFLD status. Compared with the non-NAFLD group, the NAFLD group had a higher mean age, a higher proportion of Mexican Americans, and significantly higher mean FBG and TC levels (*P* < .05), while exhibiting substantially lower household income ratios and high-density lipoprotein levels. Conversely, the NAFLD group demonstrated significantly higher mean values for TG, LDL, uric acid, aspartate aminotransferase, alanine aminotransferase, alkaline phosphatase, C-reactive protein, WC, WHtR, BMI, TyG, TyG-WC, TyG-WHtR, TyG-BMI, TyG-WWI, and LSM (*P* < .001).

**Table 1:** Characteristics of study population.

Variables	Total	Non-NAFLD	NAFLD	*P*
N = 678	N = 563	N = 115
Age, yr	15.47 ± 2.21	15.37 ± 2.23	15.92 ± 2.07	.015
Gender, %				.545
Male	354 (52.21%)	291 (51.69%)	63 (54.78%)	
Female	324 (47.79%)	272 (48.31%)	52 (45.22%)	
Race, %				.039
Mexican American	116 (17.11%)	86 (15.28%)	30 (26.09%)	
Other Hispanic	63 (9.29%)	55 (9.77%)	8 (6.96%)	
Non-Hispanic White	217 (32.01%)	185 (32.86%)	32 (27.83%)	
Non-Hispanic Black	162 (23.89%)	132 (23.45%)	30 (26.09%)	
Other race	120 (17.70%)	105 (18.65%)	15 (13.04%)	
Smoking, %				.184
Yes	9 (1.33%)	6 (1.07%)	3 (2.61%)	
No	669 (98.67%)	557 (98.93%)	112 (97.39%)	
Income-to-poverty ratio	2.15 ± 1.49	2.26 ± 1.53	1.60 ± 1.14	<.001
FPG	97.97 ± 18.56	96.82 ± 7.78	103.63 ± 41.33	.004
TG (mg/dL)	69.83 ± 41.95	64.88 ± 36.97	94.07 ± 54.75	<.001
TC (mg/dL)	153.71 ± 29.26	152.02 ± 28.62	162.00 ± 31.02	.002
HDL (mg/dL)	52.15 ± 11.72	53.47 ± 11.69	45.70 ± 9.62	<.001
LDL-C (mg/dL)	87.62 ± 25.65	85.61 ± 24.87	97.45 ± 27.19	<.001
UA	5.11 ± 1.25	4.99 ± 1.19	5.67 ± 1.39	<.001
AST	20.34 ± 14.36	20.16 ± 15.44	21.24 ± 6.92	<.001
ALT	17.05 ± 19.30	15.57 ± 19.71	24.31 ± 15.26	<.001
ALP	142.29 ± 92.40	144.53 ± 95.54	131.34 ± 74.54	.518
CRP	2.21 ± 5.58	1.88 ± 5.50	3.83 ± 5.70	<.001
WC (cm)	83.52 ± 16.52	79.26 ± 12.70	104.36 ± 17.23	<.001
WHtR	0.50 ± 0.10	0.48 ± 0.08	0.62 ± 0.10	<.001
BMI (kg/m^2^)	24.92 ± 6.98	23.23 ± 5.36	33.18 ± 8.02	<.001
WWI	10.14 ± 0.80	9.99 ± 0.73	10.88 ± 0.72	<.001
TyG	7.99 ± 0.55	7.92 ± 0.51	8.31 ± 0.60	<.001
TyG-WC	670.04 ± 155.46	629.22 ± 116.61	869.89 ± 167.20	<.001
TyG-WHtR	4.05 ± 0.93	3.81 ± 0.71	5.21 ± 0.99	<.001
TyG-BMI	200.12 ± 62.26	184.49 ± 46.41	276.66 ± 72.68	<.001
TyG-WWI	81.14 ± 9.66	79.23 ± 8.52	90.50 ± 9.48	<.001
LSM (kPa)	5.31 ± 3.44	5.02 ± 2.96	6.71 ± 4.97	<.001

ALP = alkaline phosphatase, ALT = alanine aminotransferase, AST = aspartate aminotransferase, BMI = body mass index, CRP = C-reactive protein, HDL = high-density lipoprotein, LDL-C = low-density lipoprotein, LSM = liver stiffness measurement, TC = total cholesterol, TG = triglycerides, TyG = triglycerides-glucose index, UA = uric acid, WC = waist circumference, WHtR = waist-to-height ratio, WWI = weight-adjusted waist index.

### 3.2. Multivariate logistic regression analysis of TyG-WC, TyG-WHtR, TyG-BMI, TyG-WWI, and NAFLD

Table 2 presents that in all models, TyG-WC, TyG-WHR, TyG-BMI, and TyG-WWI showed significant positive correlations with NAFLD (*P* < .001). For each additional unit of TyG-WWI, the risk of fatty liver disease increased by 16%, while each unit increase in TyG-WHtR elevated the risk 6-fold (odds ratio = 6.03, 95% CI: 3.82–9.52).

**Table 2:** Multivariate logistic regression analysis of TyG-WC, TyG-WHtR, TyG-BMI, TyG-WWI, and NAFLD.

Exposure	Non-adjusted	Adjust I	Adjust II
	β (95% CI), *P* value	β (95% CI), *P* value	β (95% CI), *P* value
TyG-WWI	1.14 (1.11–1.18), <.0001	1.16 (1.13–1.20), <.0001	1.16 (1.11–1.21), <.0001
TyG-WHtR	5.52 (4.09–7.45), <.0001	5.95 (4.33–8.16), <.0001	6.03 (3.82–9.52), <.0001
TyG-WC	1.01 (1.01–1.01), <.0001	1.01 (1.01–1.01), <.0001	1.01 (1.01–.01), <.0001
TyG-BMI	1.02 (1.02–1.03), <.0001	1.03 (1.02–1.03), <.0001	1.02 (1.02–1.03), <.0001

Model 1: no covariates were adjusted. Model 2: age, gender, race, and energy intake were adjusted. Model 3: age, gender, race, income-to-poverty ratio, smoking status, LDL-C, HDL, UA, AST, ALT, ALP, LSM, and CRP were adjusted.

ALP = alkaline phosphatase, ALT = alanine aminotransferase, AST = aspartate aminotransferase, CRP = C-reactive protein, HDL = high-density lipoprotein, LDL-C = low-density lipoprotein, LSM = liver stiffness measurement, NAFLD = nonalcoholic fatty liver disease, TyG-BMI = triglyceride glucose–body mass index, TyG-WC = triglyceride glucose–waist circumference, TyG-WHtR = triglyceride glucose–waist-to-height ratio, TyG-WWI = triglyceride-glucose weight-adjusted waist index, UA = uric acid.

### 3.3. Interaction test for smooth curve fitting of the correlation between TyG-WC, TyG-WHtR, TyG-BMI, TyG-WWI, and NAFLD

**Figure 2.**
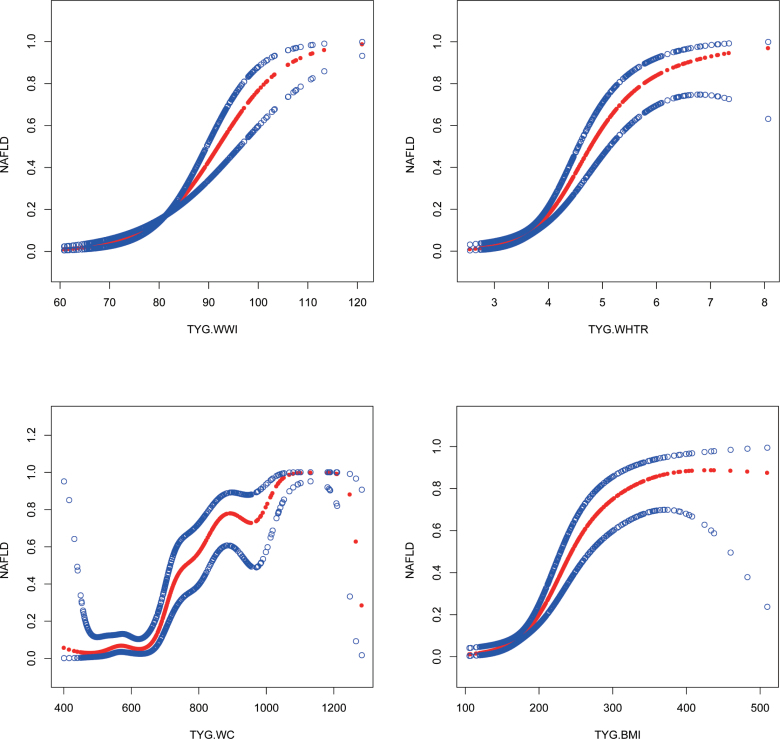
Smooth curve fitting of the correlation between TyG-WC, TyG-WHtR, TyG-BMI, TyG-WWI, and NAFLD. NAFLD = nonalcoholic fatty liver disease, TyG-BMI = triglyceride glucose–body mass index, TyG-WC = triglyceride glucose–waist circumference, TyG-WHtR = triglyceride glucose–waist-to-height ratio, TyG-WWI = triglyceride-glucose weight-adjusted waist index.

Figure 2 shows that NAFLD occurs very rarely when TyG-WC values fall within the 400 to 700 range, TyG-WHtR values within the 2 to 4 range, TyG-BMI values within the 100 to 200 range, and TyG-WWI values within the 60 to 80 range. When TyG-WWI >80 cm/√kg, TyG-WHtR >4, TyG-WC >700 cm, or TyG-BMI >200 kg/m^2^, the risk of NAFLD significantly increases. Table 3 indicates that gender shows no statistically significant difference in the relationships between TyG-WC, TyG-WHtR, TyG-WWI, and NAFLD. Similarly, ethnicity shows no statistically significant difference in the interaction tests between these variables.

**Table 3:** Interaction test for the relationship between TyG-WC, TyG-WHtR, TyG-BMI, TyG-WWI, and NAFLD.

	TyG-WC	*P*	TyG-WHtR	*P*		TyG-BMI	*P*	TyG-WWI	*P*
Gender		.1621		.1529			.0455		.6300
Male	1.01 (1.01–1.02), <.0001		8.94 (4.56–17.54), <.0001		1.03 (1.02–1.04), <.0001			1.17 (1.11–1.24), <.0001	
Female	1.01 (1.01–1.01), <.0001		5.13 (2.98–8.83), <.0001		1.02 (1.01–1.03), <.0001			1.15 (1.09–1.22), <.0001	
Race		.0317		.4370			.4072		.3404
Mexican American	1.01 (1.00–1.02), .0071		5.93 (1.57–22.37), .0087		1.03 (1.01–1.05), .0079			1.12 (0.98–1.27), .0934	
Other Hispanic	1.02 (1.00, 1.04), .0845		10.39 (0.67–161.14), .0941		1.04 (0.99–1.09), .0978			1.06 (0.84–1.33), .6357	
Non-Hispanic White	1.02 (1.01–1.03), <.0001		19.01 (5.94–60.86), <.0001		1.04 (1.02, 1.05), <.0001			1.31 (1.15–1.49), <.0001	
Non-Hispanic Black	1.01 (1.01–1.02), <.0001		6.09 (2.57–14.42), <.0001		1.02 (1.01–1.03), .0002			1.20 (1.10–1.32), <.0001	
Other race	1.02 (1.00–1.04), .0244		30.90 (1.53–622.80), .0252		1.05 (1.01–1.09), .0211			1.18 (0.96–1.45), .1175	

Age, income-to-poverty ratio, smoking status, LDL-C, HDL, UA, AST, ALT, ALP, LSM, and CRP were adjusted.

ALP = alkaline phosphatase, ALT = alanine aminotransferase, AST = aspartate aminotransferase, CRP = C-reactive protein, HDL = high-density lipoprotein, LDL-C = low-density lipoprotein, LSM = liver stiffness measurement, NAFLD = nonalcoholic fatty liver disease, TyG-BMI = triglyceride glucose–body mass index, TyG-WC = triglyceride glucose–waist circumference, TyG-WHtR = triglyceride glucose–waist-to-height ratio, TyG-WWI = triglyceride-glucose weight-adjusted waist index, UA = uric acid.

### 3.4. Diagnostic performance of TyG-WC, TyG-WHtR, TyG-BMI, and TyG-WWI for NAFLD

**Figure 3.**
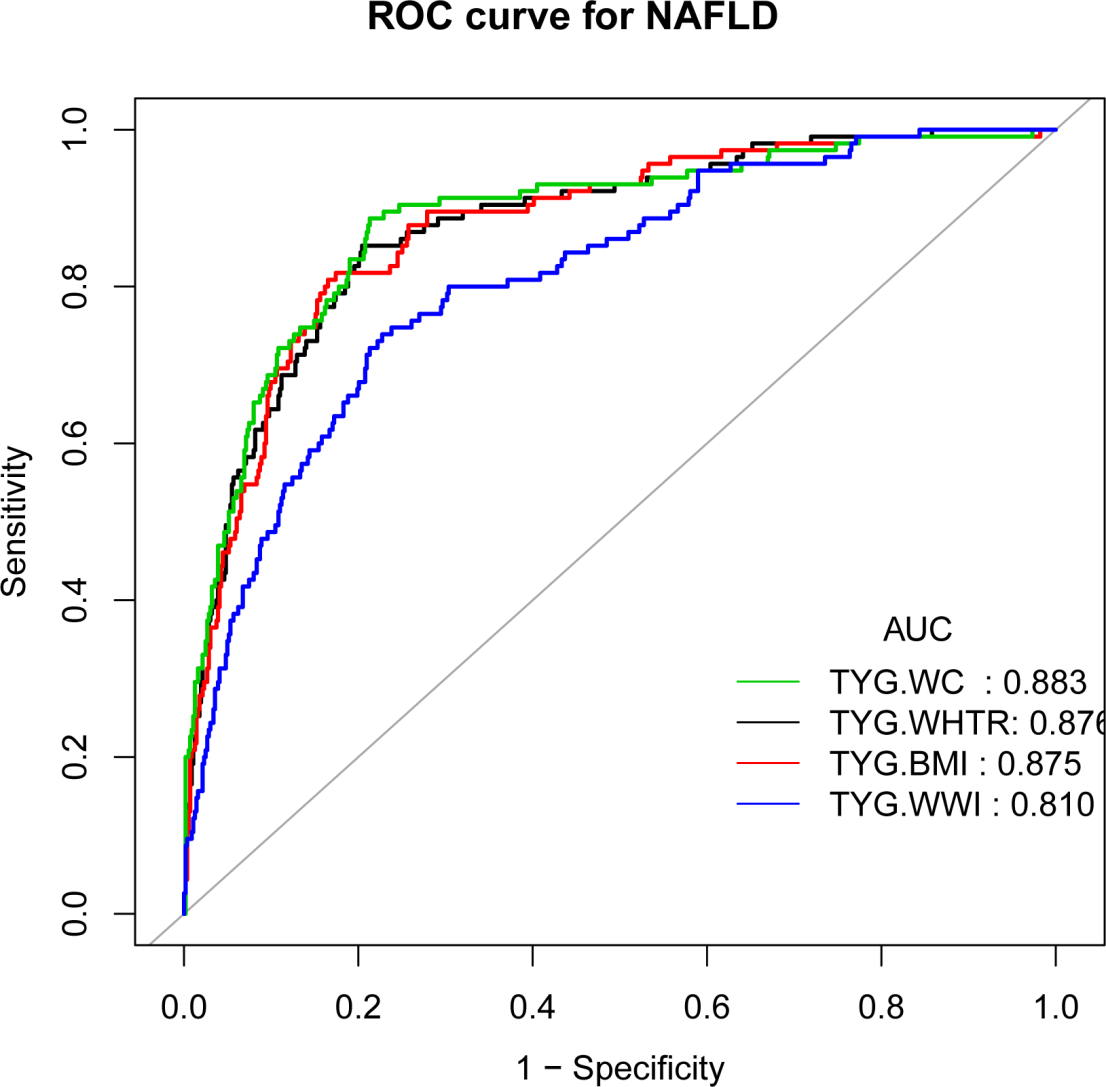
ROC for the correlation between TyG-WC, TyG-WHtR, TyG-BMI, TyG-WWI, and NAFLD. AUC = area under the curve, NAFLD = nonalcoholic fatty liver disease, ROC = receiver operating characteristic, TyG-BMI = triglyceride glucose–body mass index, TyG-WC = triglyceride glucose–waist circumference, TyG-WHtR = triglyceride glucose–waist-to-height ratio, TyG-WWI = triglyceride-glucose weight-adjusted waist index.

Figure 3 demonstrates that the TyG-WC, TyG-WHtR, TyG-BMI, and TyG-WWI indices all exhibit good diagnostic value for NAFLD, with area under the curve values exceeding 0.8. Among these, TyG-WC showed the optimal diagnostic performance, with the highest area under the curve value (0.8833) for TyG-waist circumference (TyG-WC). Table 4 indicates that TyG-WC demonstrated the highest sensitivity (88.70%). The negative predictive value of TyG-WC was 97.15%, while its positive predictive value was 45.95%.

**Table 4:** Diagnostic performance of TyG-WC, TyG-WHtR, TyG-BMI, and TyG-WWI for NAFLD.

Index	AUC	Cutoff 95% CI	Best threshold	Specificity (%)	Sensitivity (%)	PPV (%)	NPV (%)
TyG-WWI	0.8101	0.7673–0.8530	85.6623	0.7726	0.7391	0.3991	0.9355
TyG-WHtR	0.8761	0.8410–0.9112	4.2947	0.7957	0.8522	0.4601	0.9634
TyG-WC	0.8833	0.8475–0.9192	703.1483	0.7869	0.8870	0.4595	0.9715
TyG-BMI	0.8748	0.8393–0.9103	220.0576	0.8348	0.8087	0.5000	0.9553

AUC = area under the ROC curve, NPV = negative predictive value, NAFLD = nonalcoholic fatty liver disease, PPV = positive predictive value, ROC = receiver operating characteristic, TyG-BMI = triglyceride glucose–body mass index, TyG-WC = triglyceride glucose–waist circumference, TyG-WHtR = triglyceride glucose–waist-to-height ratio, TyG-WWI = triglyceride-glucose weight-adjusted waist index.

## 4. Discussion

Based on the study population aged 12 to 19 years, the following findings were observed: compared with the non-NAFLD group, the NAFLD group exhibited poorer family economic conditions in terms of external objective factors; significant correlations between TyG-WC, TyG-WHtR, TyG-BMI, and TyG-WWI and NAFLD risk; NAFLD risk was significantly lower when TyG-WC, TyG-WHtR, TyG-BMI, and TyG-WWI were <700 cm, <4, <200 kg/m^2^, and <80 cm/√kg, respectively; and for predicting adolescent NAFLD disease status, TyG-WHtR, TyG-BMI, and TyG-WWI, particularly TyG-WC, demonstrated superior diagnostic value.

The pathogenesis of NAFLD is considered a complex interplay involving insulin resistance, excessive hepatic TG accumulation, genetic factors, gut microbiota dysbiosis, inflammatory responses, hormonal fluctuations during adolescence, as well as dietary and exercise habits.^[20]^ Adolescents undergo significant hormonal changes during puberty, accompanied by noticeable shifts in body shape as they grow in height and weight. Current research indicates that the prevalence of NAFLD among obese populations is increasing year by year.^[21]^ However, NAFLD also occurs in nonobese individuals. We believe certain threshold ranges may exist that facilitate early intervention and diagnosis. TG-glucose-derived obesity indices serve as metabolic markers that combine insulin resistance with obesity. By controlling TyG-WC, TyG-WHtR, TyG-BMI, and TyG-WWI thresholds, we aim to improve insulin resistance and hepatic steatosis, thereby reducing NAFLD prevalence.^[22]^ Obesity-induced fat accumulation leads to adipocyte hypertrophy, hyperplasia, and dysfunction. Reduced insulin-mediated inhibition of lipolysis in fat cells triggers lipolysis, releasing large amounts of free fatty acids.^[23]^ These fatty acids flood into the liver, causing hepatic TG synthesis to exceed the hepatocyte’s oxidative capacity, resulting in the deposition and formation of fatty liver. Insulin resistance in the liver manifests as impaired inhibition of gluconeogenesis by insulin, while pathways promoting lipid synthesis may even be enhanced.^[24]^ Hyperinsulinemia impairs hepatic fatty acid oxidation pathways, further exacerbating TG accumulation in the liver. Obesity induces cell apoptosis and dysregulation of adipokines, triggering inflammatory responses that transform simple fatty liver into steatohepatitis.^[25,26]^

The findings of this study indicate that the NAFLD group exhibited poorer household economic conditions, consistent with the view presented by Yuandong Luo, Meiling Tang, and colleagues that the rising prevalence of NAFLD among adolescents is negatively correlated with economic conditions in the United States.^[27,28]^ Research by Guotai Sheng et al suggests that TyG-BMI and TyG-WC may serve as good predictors of NAFLD.^[29]^ This study excluded TyG as an independent factor and incorporated the TyG-WWI index to reflect central obesity (visceral fat accumulation), confirming the stability of results through interaction testing. Other studies indicate that racial factors influence the prevalence of NAFLD.^[30]^ However, this study yielded opposite results, which may be related to the fact that all subjects in this study were aged 12 to 19 years. Jeffrey et al found that a low-free-sugar diet improved hepatic steatosis in adolescent boys aged 11 to 16 years.^[31]^ This indicates that healthy eating is effective. Weight loss achieved through calorie restriction is central to improving NAFLD. A daily reduction in calorie intake – regardless of specific dietary composition – leading to 5% to 10% weight loss can improve NAFLD.^[32,33]^ For example, the following 4 categories: low-carbohydrate diets, where carbohydrates account for <45% of total daily calories; ketogenic diets (extremely low carbohydrates, high fat, moderate protein); Mediterranean diets (olive oil as primary fat source, limited intake of red meat, processed meats, and sweets); and low-fructose diets (reducing sugary beverages and processed foods containing syrups).^[34]^ Exercise enhances muscle absorption and fatty acid oxidation, which decreases the storage of hepatic fat and aids in weight loss.^[35]^ It reduces circulating insulin levels, improves insulin resistance, and increases muscle glucose absorption and glycogen synthesis. It lowers the incidence of NAFLD by inhibiting the synthesis of hepatic inflammatory factors.^[36,37]^

This study has several limitations, including the inability to establish causality and the need for further investigation into its clinical utility for adolescent prevention and diagnosis. Although many confounding factors were accounted for, the potential influence of additional minor factors cannot be entirely ruled out. The strengths of this study lie in its large sample size, which enhances reliability and national representativeness, as well as its rigorous methodology. Detailed anthropometric measurements were combined with laboratory data. While liver biopsy is considered the gold standard for diagnosing NAFLD, noninvasive methods such as liver ultrasound and transient elastography are increasingly recognized as valid and preferred approaches. This study provides a reference for future research on potential therapeutic or preventive strategies for adolescent NAFLD, including maintaining obesity indicators derived from TyG within effective ranges and achieving therapeutic efficacy.

This study suggests a link between simplistic obesity-derived indices with NAFLD, which can provide a cost-effective strategy to identify NAFLD early in adolescents, allowing for better detection and subsequently improved management and treatment. This study focuses on American adolescents during a specific developmental period. Differences from other research may stem from the unique characteristics of this age group, which is in a peak phase of growth and development. TG-glucose-derived obesity indices can serve as screening tools; this study incorporated TyG-WWI, an indicator potentially better reflecting visceral fat dynamics. The core insulin resistance underlying NAFLD is not solely related to body weight. Future cohort studies may be considered to clarify causal relationships.

## 5. Conclusion

TyG-WC, TyG-WHtR, TyG-BMI, and TyG-WWI are significantly associated with NAFLD risk in adolescents aged 12 to 19 years and may hold clinical value for predicting NAFLD in this population.

## Acknowledgments

We express our appreciation to the participants and staff of the NHANES project.

## Author contributions

**Conceptualization:** Wenting Deng, Jian Mei, Aiguo Wang.

**Data curation:** Wenting Deng, Bichao Yu.

**Formal analysis:** Wenting Deng.

**Methodology:** Wenting Deng, Qingmin Yu, Weixin Pan.

**Software:** Guangli Ding.

**Supervision:** Hongli Zhou.

**Visualization:** Ashanjiang Aniwan.

**Writing – original draft:** Wenting Deng.

**Writing – review & editing:** Wenting Deng.
